# Anesthetic management of cesarean section in a patient with a large anterior mediastinal mass: a case report

**DOI:** 10.1186/s40981-017-0098-1

**Published:** 2017-05-10

**Authors:** Kunio Kusajima, Satoshi Ishihara, Takeshi Yokoyama, Katsuyuki Katayama

**Affiliations:** Department of Anesthesiology, Teine Keijinkai Hospital, 1-40 1-jo 12-chome, Maeda, Teine-ku, Sapporo, Hokkaido 006-8555 Japan

**Keywords:** Anesthetic management, Mediastinal mass, Pregnancy, Cesarean section, Tracheal stenosis, Respiratory distress, Combined spinal epidural anesthesia

## Abstract

**Background:**

Symptomatic anterior mediastinal mass in pregnancy is rare, and cesarean section for such patients poses a risk of cardiopulmonary collapse.

**Case presentation:**

A 30-year-old woman at 40 weeks’ gestation complained of breathlessness and cough, and she was not able to lie supine because of respiratory distress. Computed tomography scan revealed a large anterior-superior mediastinal mass severely compressing the trachea, bilateral main bronchus, and superior vena cava. Because clinical symptoms and computed tomographic findings suggested imminent respiratory catastrophe, urgent cesarean section was planned. The patient was able to lie in the semi-recumbent position with minimal symptoms; therefore, we considered it safe to perform cesarean section with combined spinal epidural anesthesia. In the event of cardiopulmonary collapse, emergent intubation and extracorporeal membrane oxygenation were also planned. The operation was performed successfully with combined spinal epidural anesthesia. The infant was healthy, and the postoperative hospital course was uneventful.

**Conclusions:**

Combined spinal epidural anesthesia is preferable in the anesthetic management of cesarean section with symptomatic anterior mediastinal mass. A well-designed preoperative strategy can lead to favorable outcomes even in this complicated situation.

## Background

Symptomatic anterior mediastinal mass in pregnancy is rare [1]. Signs and symptoms are numerous and usually include cough, chest pain, dyspnea, hoarseness, orthopnea, superior vena cava syndrome, syncope, and dysphagia [2]. Cesarean section for such patients poses a risk of cardiopulmonary collapse because of compression of the tracheobronchial tree and/or great vessels. Patient management requires a thorough understanding of the pathophysiological changes caused by not only pregnancy but also the mediastinal mass. It is not clear whether general anesthesia or regional anesthesia is best for cesarean delivery in this situation [1–5].

We present a case of a pregnant woman with a mediastinal mass and associated respiratory distress who underwent cesarean section successfully with combined spinal epidural anesthesia (CSEA).

## Case presentation

**Fig. 1:**
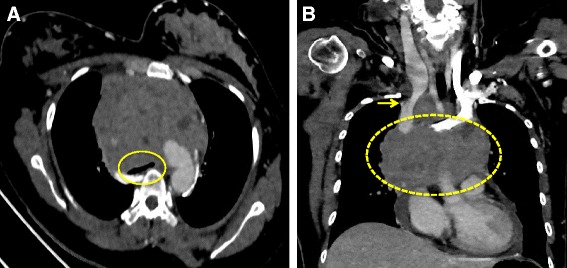
**a** CT scan showing the mediastinal mass severely compressing the trachea (*solid line circle*). **b** Enhanced CT scan showing a large anterior-superior mediastinal mass [113 × 87 × 68 mm (length, height, width; *dotted line circle*)] compressing the superior vena cava (*yellow arrow*)

A 30-year-old woman (150 cm, 61 kg) at 40 weeks’ gestation complained of breathlessness and cough. The patient was apparently asymptomatic until 31 weeks’ gestation when she developed progressive breathlessness and cough and was unable to lie supine because of the respiratory distress. Oxygen saturation level was 94% on room air in the upright sitting position and decreased to 91% in the supine position. Blood pressure and heart rate remained stable. Chest radiographs revealed mediastinal enlargement and narrowed tracheobronchial tree, and enhanced computed tomography (CT) scan revealed a large anterior-superior mediastinal mass measuring 113 × 87 × 68 mm (length, height, width). The mass severely compressed the trachea [minimum diameter, 3.5 mm], bilateral main bronchus, and the superior vena cava (SVC) (Fig. 1a, b).

Because clinical symptoms and CT findings suggested imminent respiratory catastrophe, urgent cesarean section followed by further evaluation and treatment of the mediastinal tumor was planned. The patient was able to lie in the semi-recumbent position (20° head-up tilt) for minutes with minimal symptoms; therefore, we considered it safe to perform cesarean section with regional anesthesia rather than general anesthesia. We planned CSEA without airway management and/or mechanical ventilation. In the event of cardiopulmonary collapse, emergent use of extracorporeal membrane oxygenation (ECMO) was also planned.

In right lateral decubitus position, an epidural catheter was inserted at the L1–L2 interspace using 17-gauge Tuohy needle. Lumbar puncture was performed at the L3–L4 interspace, and 10 mg of 0.5% hyperbaric bupivacaine with 20 μg of fentanyl was injected using a 27-gauge pencil-point spinal needle. Sensory loss below the dermatome of the fourth thoracic level was achieved. After CSEA was initiated, hemodynamics remained stable, so ECMO was less likely to be established. We just sterilized the bilateral inguinal region for femoral vessels access in case of an emergency requirement for ECMO.

Fetal heart rate was being monitored concurrently and was reassuring. Throughout the procedure, the patient was able to maintain the semi-recumbent position with minimal symptoms, and oxygen saturation remained above 99% at 3 L/min oxygen with nasal cannula. The cesarean section was performed in a semi-recumbent position without difficulty and the condition of the baby was normal with APGAR scores of 8/10 and 8/10 at 1 and 5 min respectively. The time of operation and anesthesia were 45 and 79 min respectively.

**Fig. 2:**
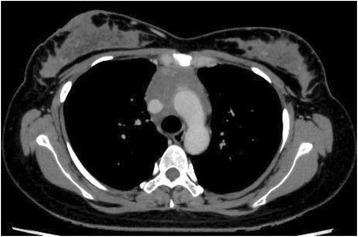
CT scan following chemotherapy showing significant regression of the mass and decreased compression of the trachea

Four hours after the operation, the patient underwent CT-guided biopsy of the mediastinal mass with local anesthesia, which led to a histological diagnosis of primary mediastinal large B cell lymphoma. The patient’s postoperative hospital course was uneventful and chemotherapy began 1 week after the operation. The lymphoma regressed significantly, and compression of the trachea, bronchus, and SVC diminished after 1 month of chemotherapy (Fig. 2).

### Discussion

Cesarean section can be performed safely under regional anesthesia in patients with cardiopulmonary compromise resulting from a large mediastinal mass. We propose that CSEA is preferable to general anesthesia in these patients.

In general, CSEA is preferable to general anesthesia for most elective cesarean sections [6, 7] because general anesthesia for cesarean section is a risk factor for mortality from pulmonary aspiration of gastric contents and failed intubation, inadequate ventilation, or both compared with CSEA, particularly in emergent situations [8]. CSEA also minimizes neonatal anesthetic exposure and estimated maternal blood loss [7, 9]. Compared with spinal anesthesia, CSEA gives a better control of the level of analgesia and can provide postoperative analgesia. Even in case spinal anesthesia fails, CSEA can help to achieve regional anesthesia successfully. So we selected CSEA rather than spinal anesthesia.

Particularly in a patient with a large mediastinal mass, general anesthesia can result in respiratory and hemodynamic collapse [10, 11]. Maintaining spontaneous respiration is effective in preventing airway collapse secondary to decreased muscle tone [12]. Also, patients with tracheal compression of more than 50% have a risk of total airway obstruction during induction of general anesthesia [13]. For these reasons, CSEA is preferable to maintain hemodynamics because if the mediastinal mass is compressing the SVC, right ventricle, and pulmonary artery, hemodynamic collapse can occur during positive pressure ventilation [10]. To predict the risk of hemodynamic collapse, preoperative cardiovascular evaluation is necessary. Diagnostic imaging studies should include CT scans to assess the degree of compression of the respiratory and cardiovascular structures, and if cardiovascular symptom appears, cardiac magnetic resonance imaging and echocardiography also should be performed [14].

Even in patients who cannot lie supine, cesarean section can be performed under CSEA by paying special attention to the patient’s positioning. In the present case, the patient could not lie supine even for 1 min, which is frequently seen in patients with severe respiratory compromise [2]. However, we found that she could lie in a semi-recumbent position for several minutes with minimal dyspnea and well-maintained arterial oxygenation. Anesthesiologists and obstetricians should carefully assess patients’ symptoms and discuss the possibility of successful cesarean section.

To safely perform cesarean section under CSEA, it is essential to plan for emergent intubation and establishing ECMO for predicted cardiopulmonary collapse [13–16]. When femoral vessel access is obtained before induction, ECMO can be established smoothly if needed [2, 3].

Delivery of the fetus can improve maternal cardiopulmonary status by relieving diaphragmatic and aortocaval compression and decreasing ventilation and cardiac output demand [6]. In the present case, respiratory distress improved significantly after cesarean section; therefore, the patient could undergo further tumor evaluation and treatment without intubation. Even in pregnant patients with a mediastinal mass and associated respiratory distress, a well-designed preoperative strategy can lead to favorable outcomes without intubation.

## Conclusions

CSEA is preferable in the anesthetic management of cesarean section with large anterior mediastinal mass. A well-designed preoperative strategy can lead to favorable outcomes even in this complicated situation.

## Abbreviations

CSEA: Combined spinal epidural anesthesia
CT: Computed tomography
ECMO: Extracorporeal membrane oxygenation
SVC: Superior vena cava
